# Supernova: A Deoxyribozyme that Catalyzes a Chemiluminescent Reaction

**DOI:** 10.1002/anie.202109347

**Published:** 2021-11-25

**Authors:** Katerina Svehlova, Ondřej Lukšan, Martin Jakubec, Edward A. Curtis

**Affiliations:** ^1^ Institute of Organic Chemistry and Biochemistry ASCR Prague Czech Republic; ^2^ Faculty of Science Charles University in Prague Prague Czech Republic

**Keywords:** aptazyme, catalytic DNA, chemiluminescence, deoxyribozyme, in vitro selection, luminescence, sensor

## Abstract

Functional DNA molecules are useful components in nanotechnology and synthetic biology. To expand the toolkit of functional DNA parts, in this study we used artificial evolution to identify a glowing deoxyribozyme called Supernova. This deoxyribozyme transfers a phosphate from a 1,2‐dioxetane substrate to its 5′ hydroxyl group, which triggers a chemiluminescent reaction and a flash of blue light. An engineered version of Supernova is only catalytically active in the presence of an oligonucleotide complementary to its 3′ end, demonstrating that light production can be coupled to ligand binding. We anticipate that Supernova will be useful in a wide variety of applications, including as a signaling component in allosterically regulated sensors and in logic gates of molecular computers.

## Graphical abstract

Artificial evolution was used to identify a light‐producing deoxyribozyme called Supernova. This catalyzes a chemiluminescent reaction in the presence of the 1,2‐dioxetane substrate CDP‐Star. Light production can also be coupled to ligand binding, suggesting that Supernova will be a useful addition to the toolkit of functional DNA parts.

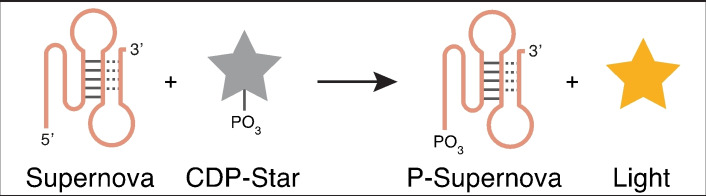

## Introduction

Following the discovery of catalytic RNA in the early 1980s,[^1^, ^2^] new methods of artificial evolution were developed to isolate nucleic acid molecules with useful and interesting properties from large random sequence pools.[^3^, ^4^, ^5^] Application of these methods revealed that both RNA and DNA molecules can catalyze diverse types of reactions, including some important in modern cells.^[6]^ These methods have also been used to generate nucleic acid motifs with the potential to facilitate basic research.[^7^, ^8^] Notable examples include a recombinase ribozyme that can insert itself into a specific target sequence,^[9]^ allosterically activated self‐cleaving ribozymes that can detect biologically important small molecules such as ATP,^[10]^ a ribozyme that covalently links a newly synthesized polypeptide chain to the mRNA that encodes it,^[11]^ an RNA motif that increases transfection efficiency,^[12]^ and aptamers that generate fluorescent signals in the presence of cognate ligands.[^13^, ^14^, ^15^]

For many applications, especially those that involve sensing, the ability to link a molecular input to an easily detectable signal is desirable. Light is a particularly useful signal for several reasons: it can be detected using widely available instruments such as plate readers, it does not introduce safety hazards such as those associated with radioactivity, and the dynamic range of such assays is typically larger than for common alternatives such as fluorescence due to a lower background.^[16]^ These considerations prompted us to investigate the possibility of creating a nucleic acid enzyme that catalyzes a chemiluminescent reaction. Here we describe the result of these studies: the development of a glowing deoxyribozyme called Supernova. This deoxyribozyme uses a 1,2‐dioxetane stabilized by a phosphate group as a substrate[^17^, ^18^] and triggers a light‐producing chemically initiated electron exchange luminescence reaction^[19]^ by transferring this phosphate from the substrate to its 5′ hydroxyl group. Comparative sequence analysis of 135,000 variants of Supernova obtained using in vitro selection and high‐throughput sequencing indicated that the catalytic core of the deoxyribozyme is made up of 38 conserved nucleotides and 46 total positions. It also revealed the unusual secondary structure of Supernova, which consists of a purine motif triple helix and two purine‐rich elements. Under optimal conditions Supernova promotes dephosphorylation of CDP‐Star 10^10^‐fold more efficiently than water. This corresponds to a rate enhancement of light production of 6,500‐fold, which far exceeds the rate enhancement of G‐quadruplex motifs that generate a chemiluminescent signal in the presence of hemin, hydrogen peroxide, and luminol.[^20^, ^21^] An engineered version of Supernova can be programmed to only generate light in the presence of an oligonucleotide complementary to its 3′ end, showing that signal production can be coupled to ligand binding. We anticipate that Supernova will be useful in a wide variety of applications, including as a signaling component in allosterically regulated sensors and in logic gates of molecular computers. Furthermore, because Supernova is made of DNA, it can be synthesized easily and cheaply, used over a wide range of conditions (including in the presence of ribonucleases), and readily optimized in various ways using the power of artificial evolution.

## Results and Discussion

**Figure 1:**
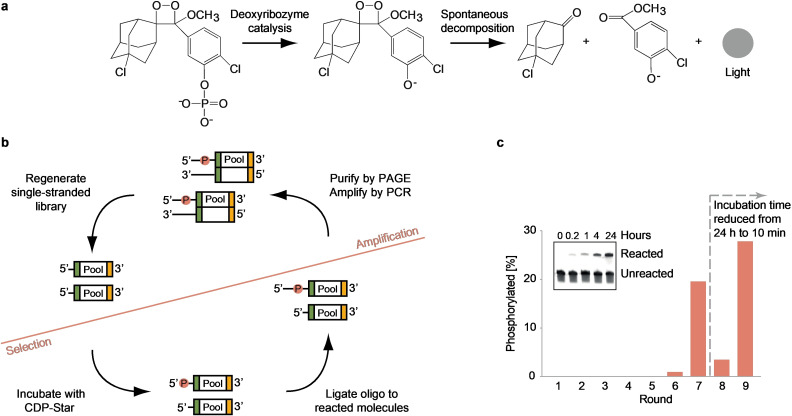
Design and progress of the in vitro selection experiment. (a) Reaction Scheme of light production using CDP‐Star. (b) Selection Scheme used to isolate deoxyribozymes that produce light by dephosphorylation of CDP‐Star. The random sequence library was first incubated with CDP‐Star. Library members that transferred a phosphate from CDP‐Star to their 5′ hydroxyl group during the incubation were tagged using a ligation reaction that requires a 5′ phosphate. These molecules were then purified by PAGE, excised from the gel, and amplified by PCR using one primer with a 5′ phosphate and one with a single ribonucleotide at the 3′ end. Single‐stranded library was then generated by incubating with lambda exonuclease (to selectively degrade one DNA strand) and base (to cleave the other DNA strand at the ribonucleotide and regenerate the 5′ end of the library). See the Supplementary Methods and Tables S1–S2 for more information. (c) Progress of the initial selection, with a time course of self‐phosphorylation of the most active deoxyribozyme identified shown in the inset.

CDP‐Star is a commercially available 1,2‐dioxetane substrate stabilized by a phosphate group, and it produces light when this phosphate group is removed.[^17^, ^18^, ^19^] CDP‐Star is typically used to detect phosphatases in solution, but can also be used in combination with oligonucleotides covalently linked to alkaline phosphatase to perform Northern Blots, or with antibodies fused to alkaline phosphatase to perform ELISA experiments. Although the chemiluminescent reaction of CDP‐Star is normally triggered by protein enzymes that hydrolyze phosphate monoesters, we hypothesized that a deoxyribozyme that transferred the phosphate group from CDP‐Star to itself would also produce light (Figure 1 a). Since a range of DNA and RNA molecules have been identified that catalyze similar phosphoryl transfer reactions,[^22^, ^23^, ^24^, ^25^, ^26^, ^27^, ^28^] the possibility that a deoxyribozyme could phosphorylate itself using CDP‐Star seemed plausible. CDP‐Star is also well‐suited as a substrate for a chemiluminescent deoxyribozyme because, unlike the light‐producing reaction of luminol catalyzed by G‐quadruplexes in the presence of hemin and hydrogen peroxide,[^20^, ^21^] the amount of background light produced in the absence of enzyme is low.

To identify DNA molecules that transfer the phosphate group of CDP‐Star to their 5′ hydroxyl group and thus trigger a light‐producing reaction, we designed a selection protocol to enrich a population for molecules containing a 5′ phosphate (Figure 1 b). In the first step of our protocol, a starting pool of 10^16^ single‐stranded DNA molecules containing a 70‐nucleotide random sequence region flanked by primer binding sites was incubated for 24 hours with CDP‐Star. A ligation reaction was then performed using pool molecules as chemically activated donors. Because DNA molecules containing a 5′ phosphate are substrates for T4 DNA ligase while those containing a 5′ hydroxyl group are not, only pool members that transferred the phosphate from CDP‐Star to their 5′‐terminus during the incubation were expected to react with the acceptor oligonucleotide. These ligation products were then isolated using PAGE and amplified by PCR using one primer which contained an RNA linkage 5′ of the phosphorylation site and a second primer which contained a 5′ phosphate. After digesting the phosphorylated strand (containing the reverse complement of the pool) with lambda exonuclease, the reaction site was regenerated by base hydrolysis to produce DNA for the next round of selection. After 7 rounds of this procedure, robust catalytic activity was detected (Figure 1 c), and after two more rounds using more stringent conditions, the pool was cloned and sequenced. Individual pool members were then synthesized and tested for catalytic activity using a ligation assay (Figure 1 c, inset). Most clones were catalytically active. More importantly, some of these deoxyribozymes also produced light (Figure S1 and Tables S3–S4), indicating that our method can be used to identify novel light‐producing deoxyribozymes.

**Figure 2:**
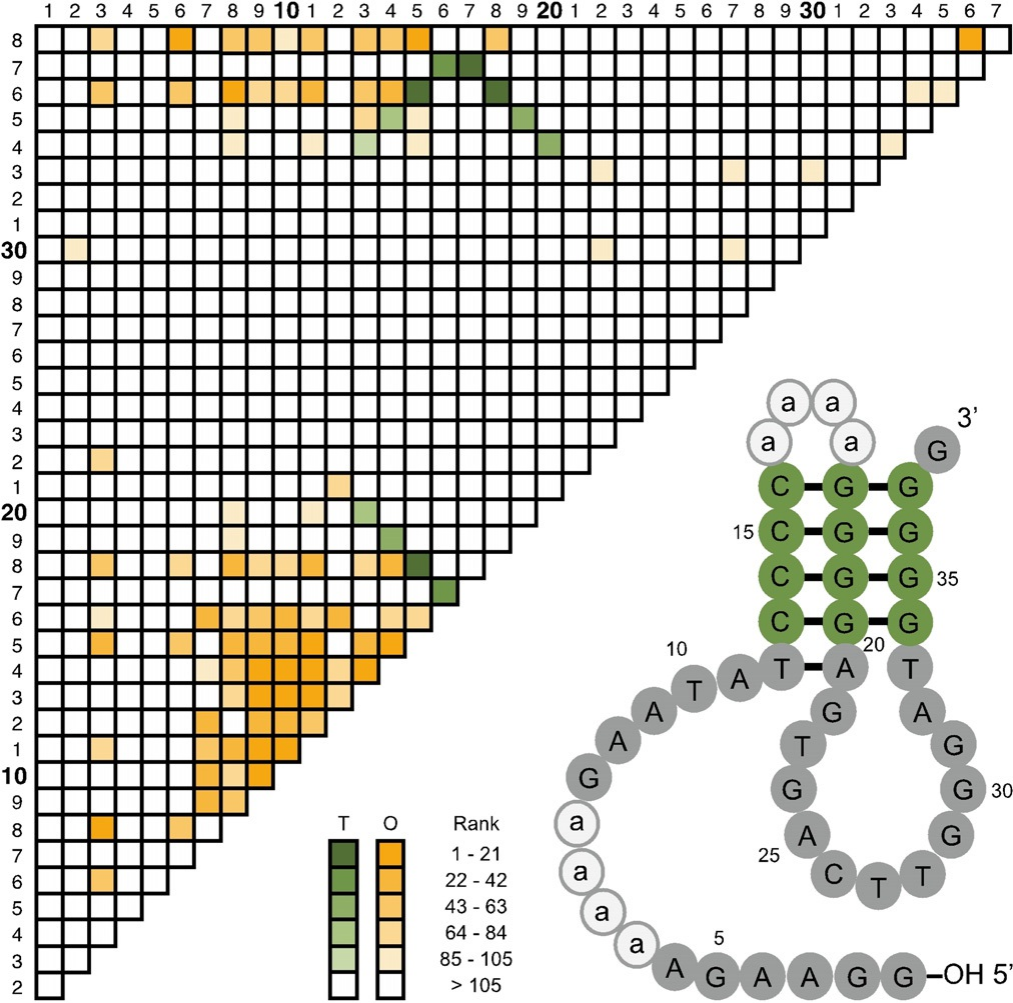
Correlation network and secondary structure of a minimized light‐producing deoxyribozyme. Correlations are ranked by mutual information value. Those corresponding to base triples (T) are shown in green, and those corresponding to other interactions (O) are shown in orange. Nucleotides in variable regions 1 and 2 are shown in lower‐case type.

To better understand the sequence requirements of these light‐producing deoxyribozymes, a second selection experiment was performed using the most efficient deoxyribozyme isolated in the initial selection as a starting point (Figure S1a). The sequence of this deoxyribozyme was randomly mutagenized at a rate of 21 % per position by chemical synthesis to generate a pool of 10^14^ deoxyribozyme variants. After six rounds of selection, activity was detected, at which point the pool was characterized using high‐throughput sequencing.^[29]^ This second selection yielded deoxyribozymes with rates of light production up to 6‐fold faster than the starting sequence (Figure S1b and Table S3). Analysis of ≈135,000 distinct deoxyribozyme variants obtained using this approach also revealed a wealth of information about the catalytic core, secondary structure, and sequence requirements of the deoxyribozyme. For example, a sequence alignment of these variants revealed three stretches of highly conserved nucleotides in the deoxyribozyme (at positions 1–6, 33–42, and 61–82) interspersed by variable regions with mutation rates similar to that of the starting pool (Figure S2). This information was used to design a minimized construct in which the variable regions at positions 7–32 (variable region 1) and 43–60 (variable region 2) were each replaced with four adenosines and positions 83–85 and the 3′ primer binding site were deleted (Figure S1, b and c). The catalytic activity of this 46‐nt mutant, which we named Supernova, was comparable to that of the full‐length sequence (Figure S1b). Comparative sequence analysis[^26^, ^30^, ^31^] also revealed a complicated network of correlated positions in the catalytic core of the deoxyribozyme (Figure 2). An unusual secondary structure model emerged from our analysis of these correlations (Figure 2). The core of our model is a triple helix capped by an 11‐nucleotide purine‐rich loop at one end and variable region 2 at the other. A second 11‐nucleotide purine‐rich motif, which is interrupted by variable region 1 and contains the phosphorylation site, is connected to the 5′ end of the triple helix. Each of the base triples in the triple helix is supported by covariations consistent with a structure in which C‐G:G, T‐A:A, and T‐A:T triples are largely interchangable, and combinations of nucleotides able to form such triples are strongly preferred at these positions (Figure S3). Such triples are characteristic of a noncanonical DNA structure called a purine‐motif triple helix, in which a purine‐rich strand forms Hoogsteen pairs with a stretch of purines in a Watson–Crick helix.[^32^, ^33^] Further evidence for this model was obtained from mutants in which proposed base triples were disrupted by single or double mutations and restored by compensatory mutations. While mutants containing disrupted triples often showed reduced activity, activity was typically restored in the compensatory mutants (Figure S3). Rates of mutants containing partially disrupted triples also correlated well with the expected number of hydrogen bonds predicted to be disrupted by these mutations (Figure S4).

**Figure 3:**
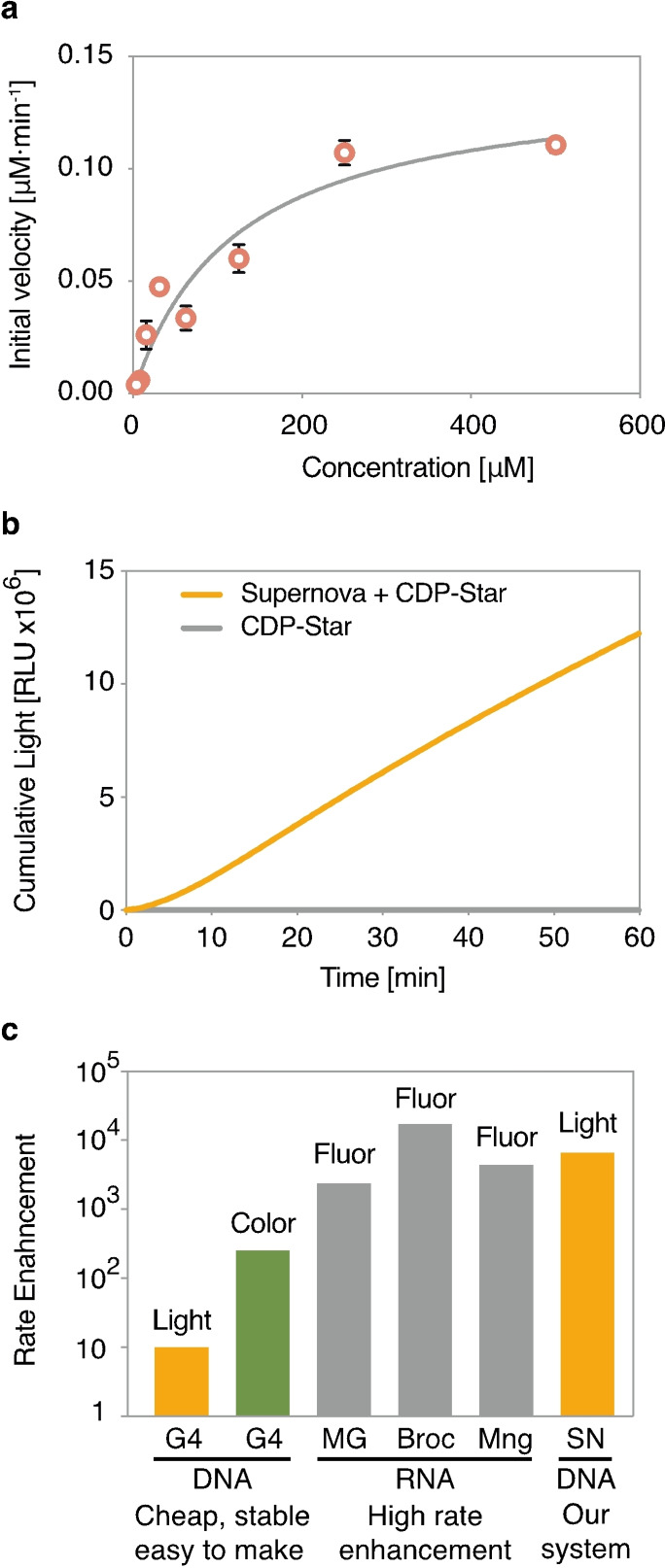
The rate of Supernova and comparison to other systems. (a) Michaelis–Menten plot showing the initial velocity of Supernova at different concentrations of substrate. Velocities were measured at a Supernova concentration of 1 μM. (b) Cumulative light production by Supernova under optimal conditions. The rate enhancement peaks at ≈6,500‐fold after 10 minutes. Reactions contained 30 μM Supernova and 62.5 μM CDP‐Star. Note that the rate at which CDP‐Star decomposes after dephosphorylation is slower than the rate of the dephosphorylation reaction catalyzed by Supernova under these conditions. For this reason, the light‐producing reaction takes longer to reach a plateau (Figure S9). (c) Comparison of Supernova to existing nucleic acid‐based methods to generate colorimetric, fluorescent, and chemiluminescent signals. G4=the DNA G‐quadruplex‐peroxidase system to generate light (in the presence of luminol) or color (in the presence of ABTS); MG=the malachite green RNA aptamer; Broc=the Red Broccoli RNA aptamer; Mng=the Mango RNA aptamer; SN=the Supernova deoxyribozyme described in this work.

The buffer used in our selection contained potassium as well as three multivalent ions (cerium, zinc, and lead) which in pilot experiment promoted the nonenzymatic hydrolysis of CDP‐Star (Figure S5). A parallel selection in the absence of these metal ions did not yield deoxyribozymes, and Supernova assays performed in buffers lacking some of them showed that only zinc was required for catalysis (Figure S6). These observations suggest a link between zinc and deoxyribozymes that utilize phosphomonoester substrates. They are also consistent with the idea that Supernova could use a mechanism similar to that of the protein enzyme alkaline phosphatase, which contains two catalytic zinc ions in the active site that interact with the hydroxyl group of the serine nucleophile, the hydrolytic water molecule, and several oxygens in the reactive phosphate of the substrate.[^34^, ^35^] Since a preferred coordination site for zinc in DNA is the N7 of guanosine, especially when it is followed by a purine,^[36]^ the requirement of Supernova for zinc could also help to explain the preponderance of highly conserved G(A/G) motifs in unpaired regions of the deoxyribozyme. Although the conservation of three of these G(A/G) motifs can be explained in part by selective pressure for library members to maintain complementarity to the constant splint sequence, this cannot account for the five additional examples which occur downstream of the splint. A CDP‐Star titration showed that Supernova follows Michaelis–Menten kinetics up to 500 μM substrate, with a *k*_cat_ of 0.15±0.03 min^−1^ and a *K*_m_ of 130±70 μM (Figure 3 a and Figure S7). Higher substrate concentrations inhibit activity, perhaps due to chelation of zinc in the buffer. Using low concentrations of substrate is also important to maximize the rate enhancement of light production, because the background amount of light produced in the absence of deoxyribozyme increases linearly from approximately 1 μM (the detection limit of the plate reader used in these experiments with respect to the nonenzymatic rate) to 3000 μM (the highest concentration tested). With the exception of CDP‐Star and the closely related CSPD, other potential substrates we tested did not support catalytic activity (Figure S8), suggesting that Supernova is relatively specific for CDP‐Star. Under optimal conditions, Supernova generates light 6,500‐fold more efficiently than the background reaction (Figures 3 b and S9), and a minimum concentration of ≈10 nM can be detected (Figure S10). Because the concentration of Supernova in these reactions (30 μM) is 10^6^‐fold lower than that of water (55 M), this corresponds to a rate enhancement of ≈10^10^‐fold. The rate enhancement of light production far exceeds that of G‐quadruplexes that generate a chemiluminescent signal in the presence of luminol or a colorimetric one in the presence of ABTS using hemin and hydrogen peroxide.[^20^, ^21^, ^37^, ^38^] It is also comparable with rate enhancements of RNA motifs that enhance the fluorescence of small‐molecule fluorophores such as the malachite green aptamer,^[13]^ Red Broccoli,^[39]^ and Mango[^15^, ^40^] (Figure 3 c). A significant advantage of our system as compared to these aptamers is that it uses DNA, which is more chemically stable, easier to prepare, and less expensive to synthesize than RNA.

**Figure 4:**
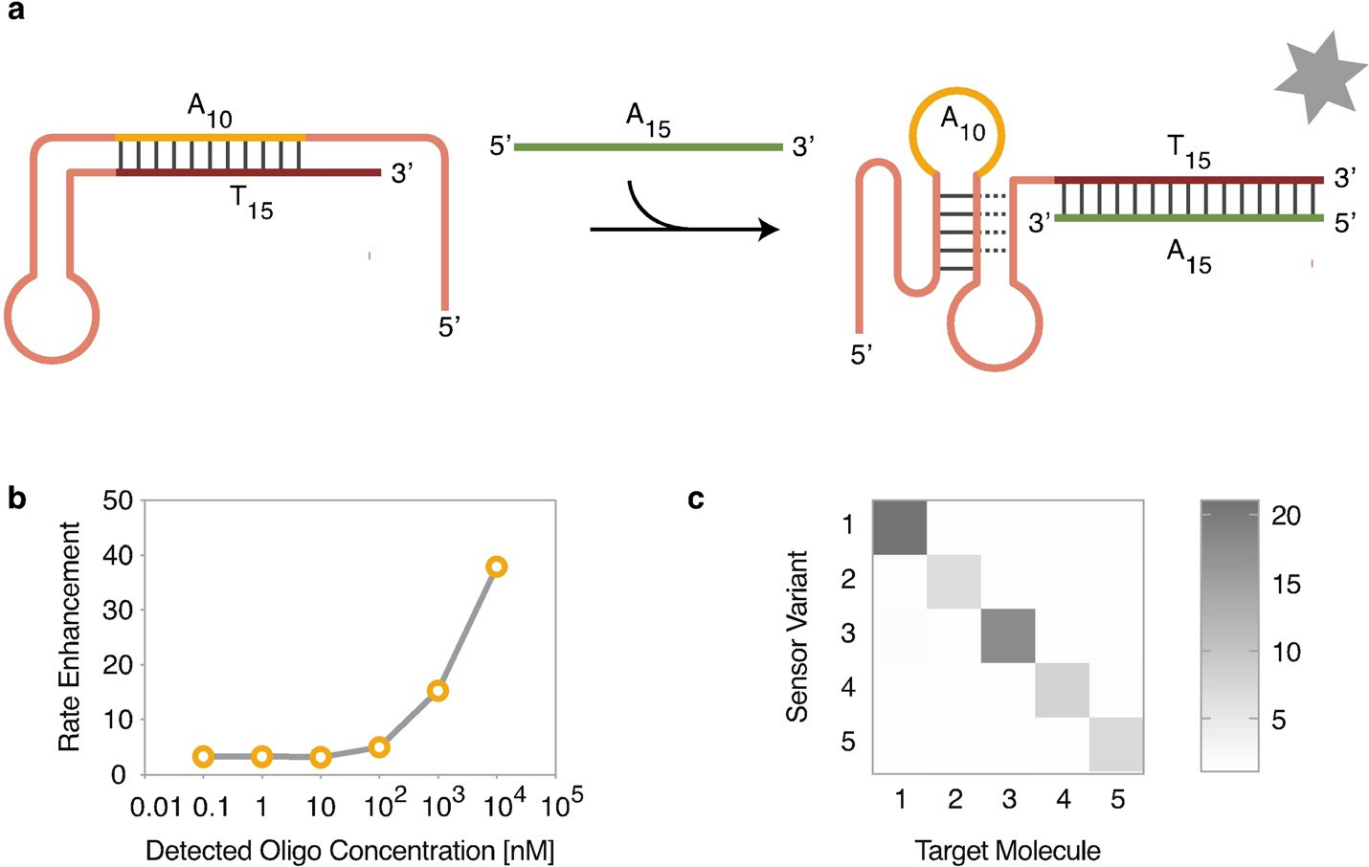
A programable light‐producing deoxyribozyme sensor that detects oligonucleotides. (a) Schematic representation of the sensor in its OFF and ON conformations. (b) Rate enhancement of light production as a function of concentration of the target oligonucleotide. (c) Rate enhancement of light production of five deoxyribozyme sensors in the presence of either the target oligonucleotide (for example, sensor 1 and oligonucleotide 1) or non‐target oligonucleotides (for example sensor 1 and oligonucleotides 2–5). The shade of each square indicates the rate enhancement of light production for a particular combination of sensor and oligonucleotide.

For many applications in DNA nanotechnology, the ability to link a molecular input, such as the binding of a ligand, to an easily detectable signal is desirable. To investigate the extent to which light production can be linked to ligand binding, we used rational design and our knowledge of the sequence requirements of Supernova to construct an allosterically regulated version of the deoxyribozyme. Our design utilized two sites (variable region 2 and the 3′ end of Supernova; Figure S1, b and c) at which insertions can be made without reducing catalytic activity. To make a light producing sensor that could detect a target oligonucleotide with a specific sequence, part of the target sequence was inserted into variable region 2 of the deoxyribozyme, while the reverse complement of the full target sequence was added to the 3′ terminus. In the absence of the target, we hypothesized that the complementary regions of variable region 2 and the 3′ end of the deoxyribozyme would hybridize and thereby prevent Supernova from adopting its catalytically active conformation (Figure 4 a). In contrast, if the target oligonucleotide was present, it would compete with variable region 2 for binding to the 3′ terminus in a concentration‐dependent manner and prevent formation of the inhibitory structure (Figure 4 a). Consistent with these predictions, Supernova produced almost no light in the absence of the target oligonucleotide, while light production increased 38‐fold in its presence. The amount of light produced was also dependent on the concentration of the target oligonucleotide (Figure 4 b), and at saturating concentrations of target the amount of light produced by the sensor was similar to that of unmodified Supernova (Figure S11). To further test the versatility of this architecture, we constructed five sensors (each specific for an oligonucleotide with a different sequence) and tested their specificity. These sensors only produced light in the presence of the oligonucleotide they were designed to detect (Figure 4 c). It is likely that the performance of these sensors can be improved by artificial evolution using a selection protocol that incorporates both negative selection (in the absence of the oligonucleotide we wish to detect) and positive selection (in the presence of the oligonucleotide we wish to detect).[^41^, ^42^, ^43^, ^44^]

We anticipate that Supernova will be useful for a wide range of applications in nanotechnology and synthetic biology. One advantage of using Supernova for such applications is that it is made of DNA, which is chemically stable, can be used over a wider range of conditions than most RNAs or proteins, and can be readily optimized in various ways using the power of artificial evolution. Another is that Supernova is small (about twice the size of a PCR primer), which means that it can be easily and inexpensively synthesized and readily incorporated into other functional DNA motifs. In addition, the chemiluminescent signal produced by Supernova can be detected using widely available instruments such as plate readers, does not introduce safety hazards such as those associated with radioactivity, and in assays has a larger dynamic range than common alternatives such as fluorescence. Furthermore, because detection is label‐free (the chemiluminescent substrate is not covalently linked to Supernova), expensive modifications of the DNA (such as fluorophores and quenchers) are not required.^[45]^ Based on these considerations, we have started to investigate the possibility that Supernova can be used to construct light‐producing sensors that are only catalytically active in the presence of a ligand of interest. As a proof of concept for this idea, we used rational design to construct a programmable version of Supernova that only produces light in the presence of an oligonucleotide with a specific sequence. By fusing Supernova to some of the many aptamers that have already been identified, it is possible that additional sensors can be constructed using this approach. A significant advantage of such sensors[^41^, ^42^, ^43^, ^44^] is that they enable detection of ligands in solution without the need for wash steps (which are used in methods such as ELISA), biochemical separations (which are required for techniques such as Northern blots), or thermocycling (which is a fundamental step in techniques such as q‐PCR). This is particularly important when speed and ease of measurement is more important than sensitivity (as is sometimes the case for applications such as point‐of‐care assays and high‐throughput screens). However, Supernova is not necessarily limited to such applications. For example, by using Supernova in combination with methods of DNA amplification such as PCR or rolling circle amplification,^[45]^ it is conceivable that higher levels of sensitivity can be achieved.

## Conclusion

Artificial evolution was used to identify a novel chemiluminescent deoxyribozyme called Supernova. The catalytic core of the deoxyribozyme is about twice the size of a PCR primer, and it forms an unusual triple helical structure. Light is generated with a rate enhancement of 6,500‐fold, and can be coupled to ligand binding. Supernova expands the toolkit of functional DNA parts, and should be useful for applications such as biosensing and molecular computing.

## Conflict of interest

The authors have applied for a patent covering the use of this light‐producing deoxyribozyme Inventors: Katerina Svehlova, Ondřej Lukšan, Martin Jakubec, and Edward A. Curtis Application number: EP21156992.6 Status: pending.

## Supporting information

As a service to our authors and readers, this journal provides supporting information supplied by the authors. Such materials are peer reviewed and may be re‐organized for online delivery, but are not copy‐edited or typeset. Technical support issues arising from supporting information (other than missing files) should be addressed to the authors.

Supporting Information
