# Supplementary material for: Supernova: A Deoxyribozyme that Catalyzes a Chemiluminescent Reaction
Source: Angew Chem Int Ed Engl. 2021 Nov 25;61(3):e202109347. doi: 10.1002/anie.202109347 (PMC9298802; doi:10.1002/anie.202109347)
Supplement: Supplementary file 1 — Supporting Information [file ANIE-61-0-s001.pdf]

Angewandte Chemie
Eine Zeitschrift der Gesellschaft Deutscher Chemiker

Supporting Information

Supernova: A Deoxyribozyme that Catalyzes a Chemiluminescent Reaction

*Katerina Svehlova, Ondřej Lukšan, Martin Jakubec, and Edward A. Curtis**

anie_202109347_sm_miscellaneous_information.pdf

Supplementary Information

This PDF file includes:

Materials and Methods
Figures S1 to S11
Tables S1 to S4

Materials and Methods

Oligonucleotides
Oligonucleotides were chemically synthesized by IDT or Sigma-Aldrich, and purified using PAGE or HPLC. See Table S1 for the sequences of all oligonucleotides used in this study.

Pool design
The initial library consisted of a random sequence region of 70 nucleotides flanked by primer binding sites of constant sequence. The sequence of the 5' end of the library was chosen based on that used in a previous selection that utilized a ligation step (Li and Breaker, 1999; ref 22 in the manuscript). Once deoxyribozymes were identified, a second library was synthesized based on the most active variant from the initial sequence (called H1; see Figure S1 and Table S1). The 5' primer binding site and the 70 nucleotides derived from the randomized region were mutated at a rate of 21% per position by chemical synthesis, while the 3' primer binding site was changed to a different constant sequence (Table S1). This was done so that, if H1 molecules in the lab contaminated the second library, they would not be amplified by PCR. To facilitate robust ligation, a splint with a constant sequence was used in the reselection (Table S1). Although this imposed a selective pressure for library variants to maintain complementarity to the splint, it did not prevent us from obtaining information about the sequence requirements of the 5' end of the deoxyribozyme: 9.3% of the H1 variants obtained in the reselection contained at least one mutation relative to H1 in this part of the sequence. We note that a strategy which makes it possible to mutagenize the 5' end of these deoxyribozymes without requiring complementarity to the splint could be useful, since the reaction site of these deoxyribozymes is the 5' hydroxyl group.

Initial Selection
The single-stranded DNA pool (Pool1) and blocking oligonucleotide (REV1) were heated in water at 65 °C for 2 minutes and cooled at room temperature for 5 minutes. They were then incubated for 24 hours (rounds 1 – 7) or 10 minutes (rounds 8 – 9) with the CDP-Star substrate (Novex) as follows: 1 μM DNA pool, 1.5 μM REV1, 1× selection buffer (50 mM HEPES pH 7.4, 200 mM KCl, 1 mM $ZnCl_2$, 1 μM $Ce(SO_4)_2$, 0.1 μM $PbCl_2$), and 1 mM CDP-Star. After the incubation, DNA was precipitated in ethanol, and ligated to a short oligonucleotide (FWD1) using a splint (Splint1) and T4 DNA ligase (Jena Bioscience). The ligation reaction was incubated for 5 minutes to reduce the likelihood of isolating kinases that use ATP rather than CDP-Star as a substrate. All oligonucleotides were at a concentration of 2.5 μM. Molecules were then separated by 6% Urea-PAGE, and those of the length 120 nt were purified. These were amplified by PCR using Taq Polymerase (Jena Bioscience) and the FWD1r and REV1 primers. The FWD1r primer contained a single RNA linkage at its 3’ end. After another ethanol precipitation, double-stranded PCR products were heated at 65 °C for 2 minutes and cooled at room temperature for 5 minutes. 10× base hydrolysis buffer was then added (1× buffer: 20 mM Trizma base, 4 M KOH, 40 mM EDTA), and the sense strands were base hydrolyzed at 90 °C for 10 minutes. The two strands were then separated by 6% Urea-PAGE to regenerate the single-stranded DNA pool. After Round 9 the pool was amplified by PCR and cloned using the TOPO TA Cloning Kit (Invirogen). Plasmids were introduced into competent *E. coli* cells (strain DH5α; Invitrogen) by chemical transformation. Plasmids from 48 selected colonies were then sequenced using Sanger sequencing (Eurofins Genomics).

Reselection
The single-stranded DNA pool (Pool2) was mixed with the blocking oligonucleotide (REV2) in water and heated at 65 °C for 2 minutes and cooled at room temperature for 5 minutes. The mixture was then incubated for 10 minutes (rounds 1 – 3) or 1 minute (rounds 4 - 7) with CDP-Star at the following final concentrations: 1 μM DNA pool, 1.5 μM REV2, 1× selection buffer, and 1 mM CDP-Star. After the incubation, DNA was ethanol precipitated and ligated to a short oligonucleotide (FWD2) using a splint (Splint2) and T4 DNA ligase (Jena Bioscience). As before, the ligation reaction was incubated for 5 minutes, and all oligonucleotides were at a concentration of 2.5 μM. Molecules were then separated by 6% Urea-PAGE, and those of the length 125 nt were purified. These were amplified by PCR using Q5 Hot Start Polymerase (NEB) and the FWD2r and REV2p primers. The FWD2r primer contained a single RNA linkage at its 3’ end and the REV2p primer contained a phosphate at its 5’ end. After cleaning up the reaction using the NucleoSpin Gel and PCR Clean-up kit (Macharey-Nagel), the reverse strand was digested using λ-exonuclease (NEB). After another purification with the NucleoSpin Gel and PCR Clean-up kit, the remaining sense strands were base-hydrolyzed and purified by 6% Urea-PAGE to regenerate the single-stranded DNA pool.

Analysis of Light Production
Oligonucleotides corresponding to individual sequences from evolved libraries were ordered from Sigma-Aldrich, and purified using 6% Urea-PAGE or HPLC. Light production was assessed as follows: the oligonucleotide being tested was mixed with the blocking oligonucleotide (if necessary) in water, heated at 65 °C for 2 minutes, and cooled at room temperature for 10 minutes. Afterwards, 5× selection buffer was added, and samples were transferred to a white half-area 96-well plate (Corning). CDP-Star was added, and chemiluminescence was immediately measured for 1 hour using a Tecan Spark plate reader (Tecan Group). Final concentrations were

1 μM of the oligonucleotide being tested, 1.5 μM of the blocking oligonucleotide, 1× selection buffer, and 1 mM CDP-Star unless stated otherwise.

Analysis of Phosphorylation
Oligonucleotides corresponding to individual sequences from evolved libraries were ordered from Sigma-Aldrich and purified by 6% Urea-PAGE. Each tested oligonucleotide was mixed with the appropriate blocking oligonucleotide (if necessary) in water, heated at 65 °C for 2 minutes and cooled at room temperature for 10 minutes. Afterwards, 5× selection buffer and CDP-Star were added. Final concentrations were 1 μM of the oligonucleotide being tested, 1.5 μM blocking oligonucleotide, 1× selection buffer, and 1 mM CDP-Star unless stated otherwise. The reaction was incubated for a specific time at room temperature in the dark and stopped by the addition of EDTA to a final concentration of 25 mM. The reaction was then cleaned up using SigmaSpin Sequencing Reaction Clean-Up columns (Sigma-Aldrich) and ethanol-precipitated. The oligonucleotide being tested was then ligated to a short oligonucleotide as in the selection except that the incubation time was 30 minutes. Reacted and unreacted molecules were then separated by 6% Urea-PAGE, and the ligation yield was analyzed using the densitometry tool ImageQuant TL (GE Healthcare LifeSciences).

Next generation sequencing and data analysis
Sequencing analysis of the randomly mutagenized pool of deoxyribozyme variants and six subsequent rounds of selection was performed on an Illumina HiSeq instrument (2×150 bp, paired-end) at GATC Biotech (Konstanz, Germany). Raw reads were processed with the cutadapt tool to remove adapter and primer sequences, perform quality trimming, and filter low quality reads. Paired-end reads were oriented, merged using the program fastq-join, and aligned using Clustal Omega. Data quality was evaluated using the FastQC tool. In each library we calculated the frequencies of unique sequences and generated sequence logos using the DiffLogo Bioconductor package. The secondary structure model was generated by mutual information analysis[30] using in-house scripts. Detailed information about all tools used is provided in Table S2.

Oligonucleotide detection using an engineered version of Supernova
The sensor variant being tested was mixed with the target oligonucleotide in water, heated at 65 °C for 2 minutes, and cooled at room temperature for 10 minutes. Afterwards, 5× optimized buffer (250 mM HEPES pH 7.4, 100 mM KCl, 5 mM $ZnCl_2$) was added, and samples were transferred to a white half-area 96-well plate (Corning). CDP-Star was added, and chemiluminescence was immediately measured for 1 hour using a Tecan Spark plate reader (Tecan Group). Final concentrations were 1 μM of the sensor, 10 μM of the target oligonucleotide, 1× selection buffer, and 250 μM CDP-Star unless stated otherwise. Light production in the absence of the target oligonucleotide was also measured for each sensor.

Kinetics measurements and analysis
Kinetics were measured using ligation assay as follows: Supernova (for the sequence see Extended Data Table 1) was mixed with water, incubated at 65 °C for 2 minutes, and cooled at room temperature for 10 minutes. Afterwards, 5× optimized buffer and CDP-Star were added. The reactions were then stopped by adding EDTA to a final concentration of 25 mM at time-points that corresponded to the linear phase of the Supernova reaction. Final concentrations were 1 μM Supernova, 1× optimized buffer, and 1 μM to 500 μM CDP-Star. Samples were then cleaned up, ethanol-precipitated, and ligations were performed as described in the section Analysis of Phosphorylation. The results were analyzed using Prism 9 software (Graphpad Software). For the calculation of k_{cat} and K_m we used the equations $\boldsymbol{V_0 = V_{max} \cdot [S]/(K_m + [S])}$ and $\boldsymbol{k_{cat} = V_{max}/[E]}$.

Calculation of rate enhancement values of other detection systems (Figure 3)
For fluorescent aptamers, rate enhancements were determined by comparing fluorescence quantum yields in the absence and presence of aptamer, and in other cases they indicate the signal in the presence of the motif divided by the signal in the absence of the motif using optimized conditions. Rate enhancements are from references 15, 37, 39, and 40.

Supplementary Figures

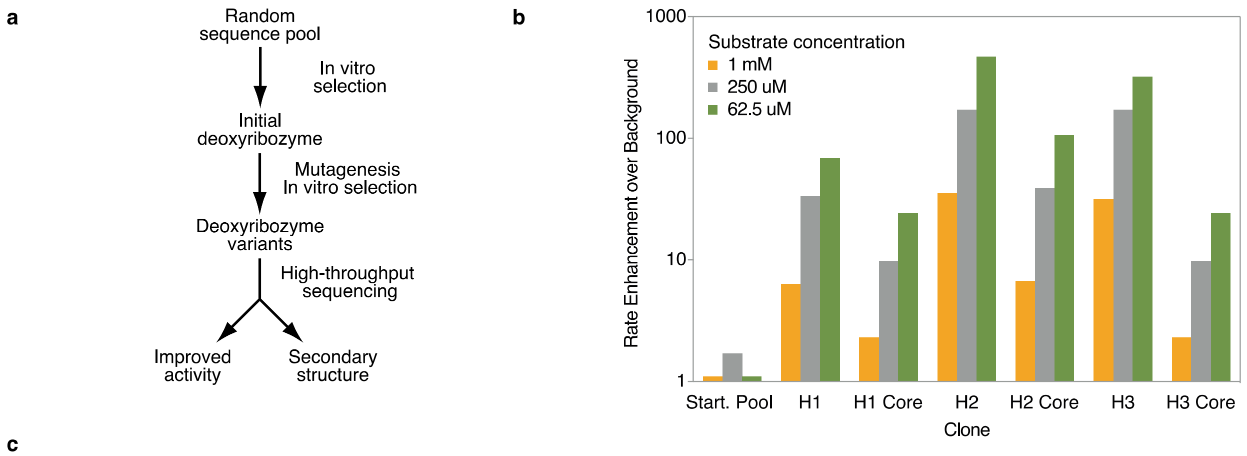

c

```
                  Variable region 1              Variable region 2                              PBS
H1        GGAAGAGATGGCGACGACACAGGGACGATGCCGAATATCCTCAGTGCGCAGGGCCGCAGGGGGGAGTGACTTGGGATGGGGGGTCAGCTGATCCTGATGG-----
H1 core   GGAAGA----------AAAA------------GAATATCCTC-------AAAA-------GGGGAGTGACTTGGGATGGGGG-----------------------
H2        GGAAGATATGGCGCGAACAAATGGACAATGCCGAATATCCCCCGCACGCAGGGCAACAAGGGGGAGTGACTTGGGATGGGGGCTGCACTAATGATCTGCCCGATG
H2 Core   GGAAGA----------AAAA------------GAATATCCCC-------AAAA-------GGGGAGTGACTTGGGATGGGGG-----------------------
H3        GGAAGAAATGTAGAGGAAACAGTGACTCTGCAGAATATCCTCACTGCGTAGTGGGGCAGGGGGGAGTGACTTGGGATGGGGGCTACACTAATGATCTGCCCGATG
H3 Core   GGAAGA----------AAAA------------GAATATCCTC-------AAAA-------GGGGAGTGACTTGGGATGGGGG-----------------------
```

Figure S1. Identification of the minimized catalytic core of Supernova. (a) Workflow of artificial evolution experiments. (b) Rate enhancement of light-production of full-length and minimized deoxyribozymes at three different concentrations of CDP-Star. Reactions contained 1 μM deoxyribozyme, 1.5 μM blocking oligonucleotide where necessary (i.e. the PBS is present) and selection buffer containing 50 mM HEPES pH 7.4, 200 mM KCl, 1 mM $ZnCl_2$, 1 μM $Ce(SO_4)_2$, 0.1 μM $PbCl_2$. (c) Sequence alignment of full-length and minimized deoxyribozymes. H1 = the most active sequence from the initial selection; H2 and H3 = two of the most abundant sequences from the reselection; H1 core, H2 core, and H3 core = minimized versions of these deoxyribozymes; variable region 1 and variable region 2 = variable regions identified by next-generation sequencing and comparative sequence analysis of evolved pools; PBS = primer binding site. Positions at which these deoxyribozymes differ from H1 are shown in pink. Note that H2 core is the sequence of Supernova.

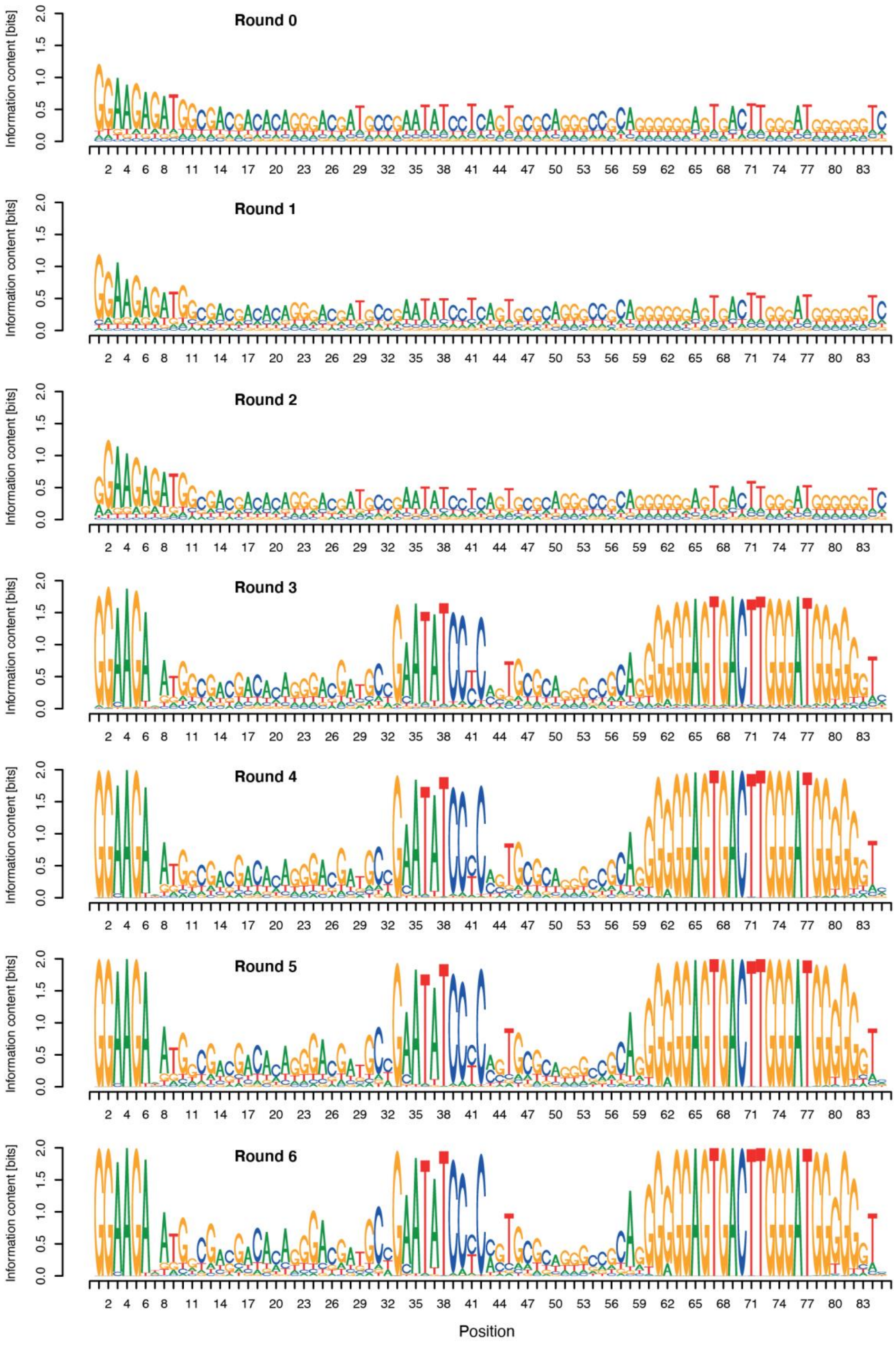
Round 0
Round 1
Round 2
Round 3
Round 4
Round 5
Round 6
Information content [bits]
0.0
0.5
1.0
1.5
2.0
2 4 6 8 11 14 17 20 23 26 29 32 35 38 41 44 47 50 53 56 59 62 65 68 71 74 77 80 83
Position

Figure S2. Identification of positions in Supernova important for catalytic activity. A library of deoxyribozyme variants was generated by randomly mutagenizing the sequence of the most active deoxyribozyme from initial selection at a rate of 21% per position. The library was characterized by high-through- put sequencing after each round of selection. The sequence logos show the extent of conservation of each position in the library after each round of selection. Positions 7-32 (variable region 1) and 43-60 (variable region 2) were replaced by AAAA spacers, and positions 83-85 as well as the 3' primer binding site (not shown) were deleted in the minimized version of the deoxyribozyme.

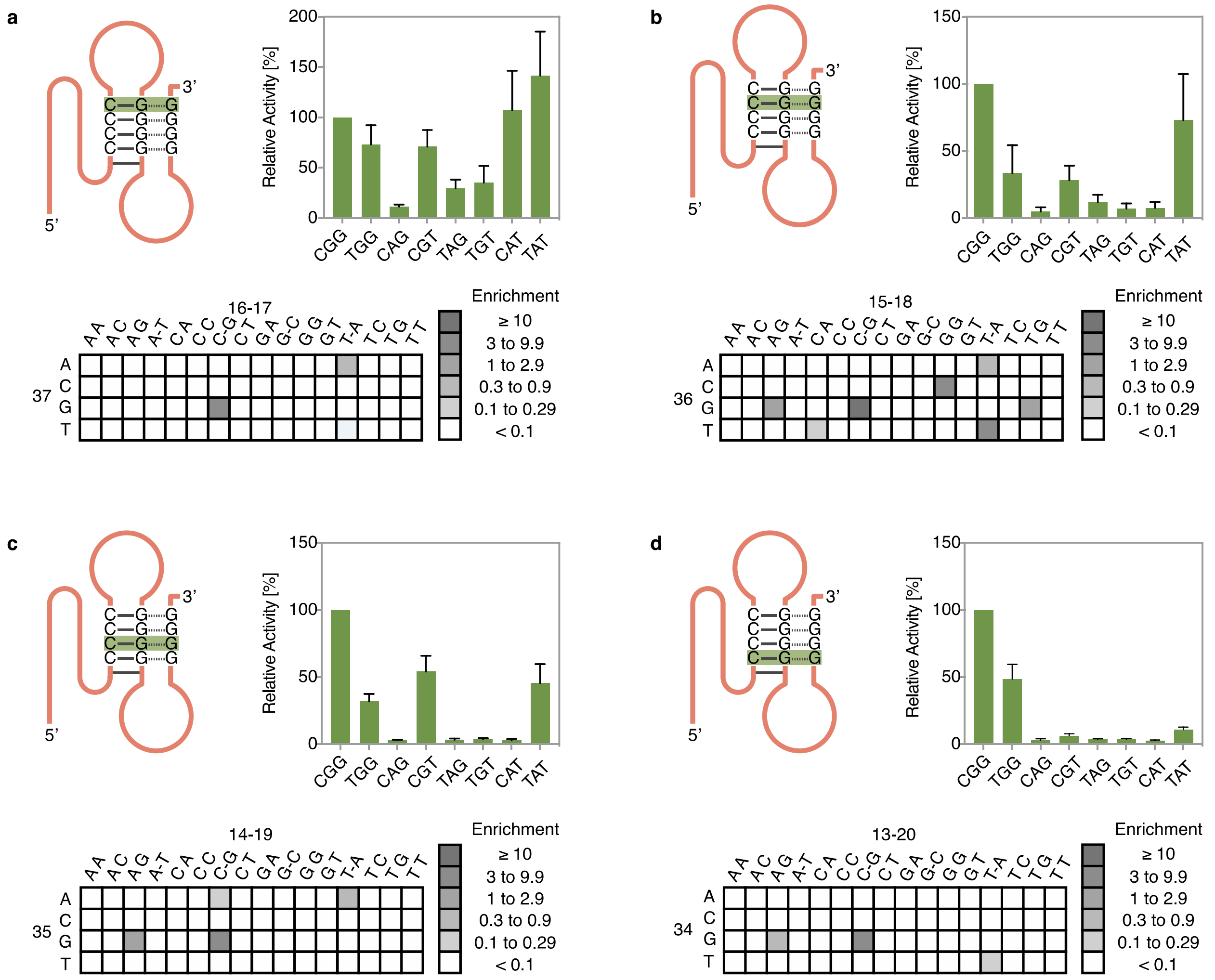

Figure S3. A purine-motif triple helix in the catalytic core of Supernova. (a) Evidence for the 16-17-37 base triple. Upper left: secondary structure model with the 16-17-37 base triple highlighted in green. Upper right: compensatory mutational effects at positions 16, 17, and 37. Activity is normalized to that of Supernova (which contains a CGG triple at this position in the triple helix). Below: nucleotide enrichments at positions 16, 17, and 37 determined by high-throughput sequencing. Enrichment values were determined by dividing the frequency of each possible sequence at positions 16, 17, and 37 in the evolved pool by its frequency in the starting pool. (b) Same, but for the 15-18-36 base triple. (c) Same, but for the 14-19-35 base triple. (d) Same, but for the 13-20-34 base triple.

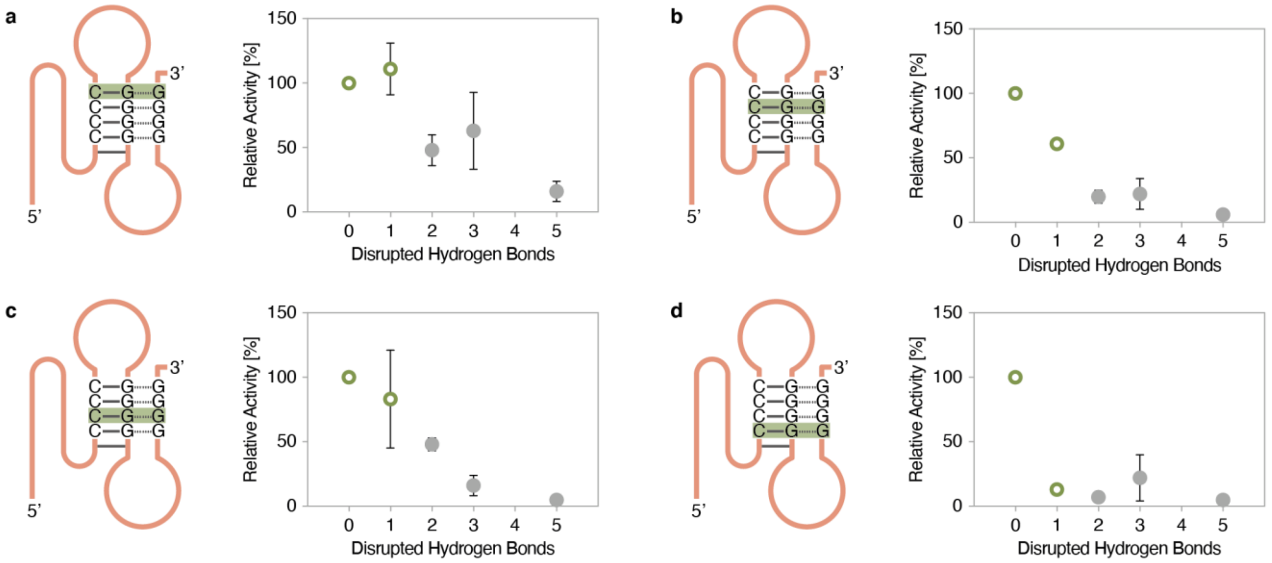

Figure S4. Relationship between the expected number of disrupted hydrogen bonds in mutated base triples and catalytic activity. This analysis assumes that each C-G G triple in the triple helix contains three hydrogen bonds on the Watson-Crick face and two hydrogen bonds on the Hoogsteen face, and that disrupting one edge of the triple does not disrupt the other (for example, two of the original five hydrogen bonds would be disrupted in a C-G A triple). (a) Activity of variants with partially or fully disrupted 16-17-37 base triple compared to the original CGG sequence. (b) Same, but for the 15-18-36 base triple. (c) Same, but for the 14-19-35 base triple. (d) Same, but for the 13-20-34 base triple. Points shown as green circles indicate canonical purine-motif base triples (C-G:G, T-A:T, or T-A:A).

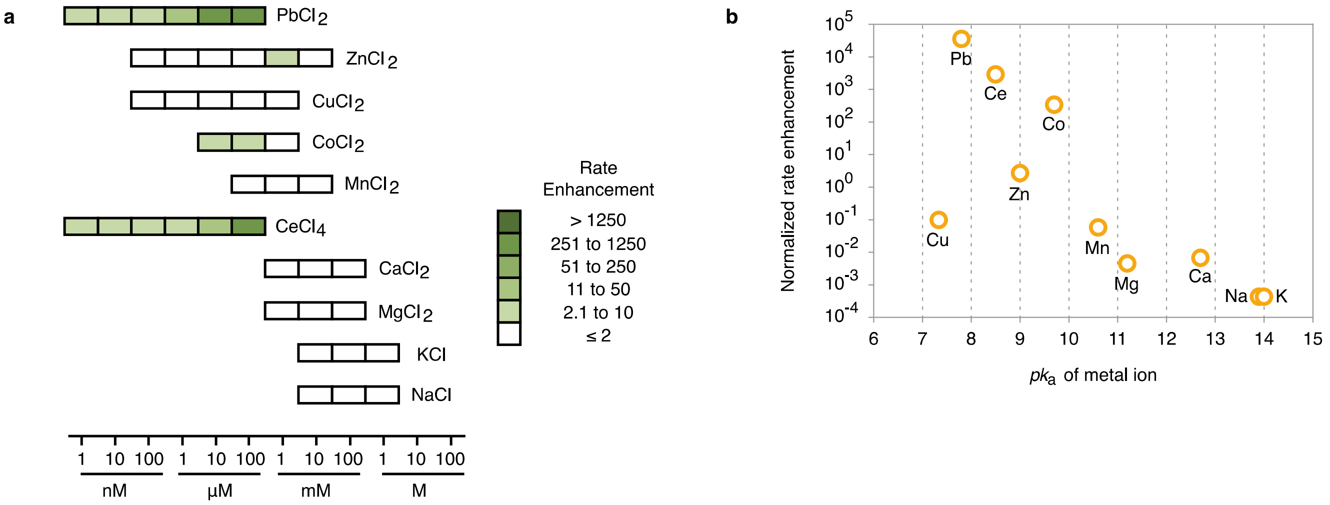

Figure S5. Effect of buffer composition on the nonenzymatic hydrolysis of CDP-Star. (a) Effects of metal ions on the nonenzymatic rate enhancement of light production by CDP-Star. The substrate was incubated in 50 mM HEPES pH 7.4 for 48 hours in the presence or absence of the indicated metal ion and light production was measured continuously. KCl, $PbCl_2$, $ZnCl_2$, and $CeCl_4$ were the metal ions in our selection buffer. A parallel selection in a buffer containing $MnCl_2$, $CaCl_2$, $MgCl_2$, KCl and NaCl did not yield deoxyribozymes. (b) Relationship between the p*K*a of the metal ion and rate enhancement of light production when normalized for concentration.

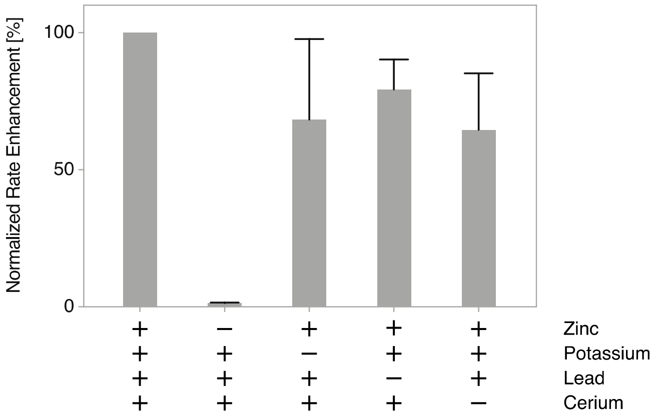

Figure S6. Metal ion requirements of Supernova. Light production of Supernova in the presence of selection buffer (50 mM HEPES pH 7.4, 200 mM KCl, 1 mM $ZnCl_2$, 1 µM $Ce(SO_4)_2$, 0.1 µM $PbCl_2$), selection buffer minus zinc, selection buffer minus potassium, selection buffer minus lead, and selection buffer minus cerium. Reactions contained 1 µM Supernova and 62.5 µM CDP-Star. Rate enhancements, which indicate the amount of light produced in the presence of deoxyribozyme divided by the amount of light produced in the absence of deoxyribozyme, were normalized to that of the reaction in the presence of each of these metal ions (++++).

[CDP-Star] = 3.9 μM

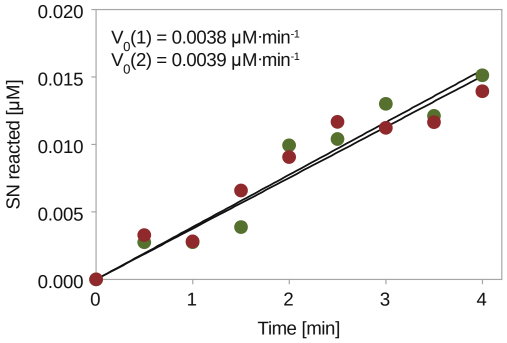

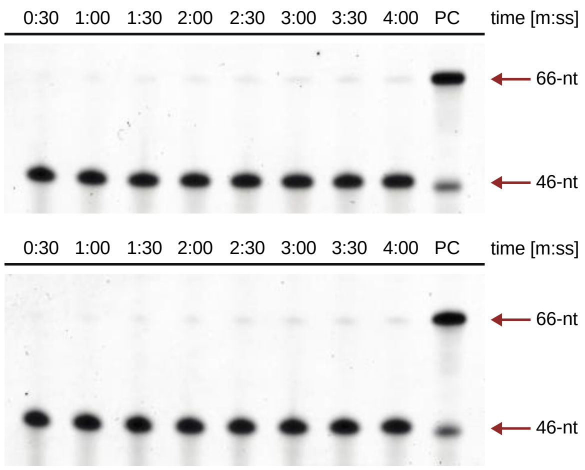

[CDP-Star] = 7.8 μM

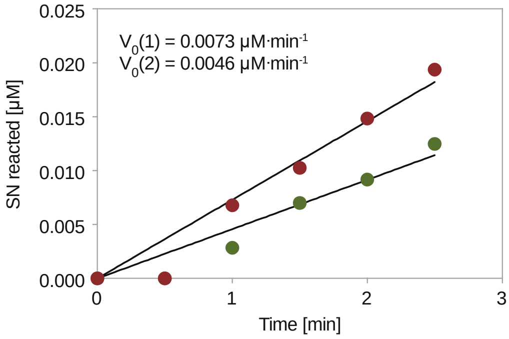

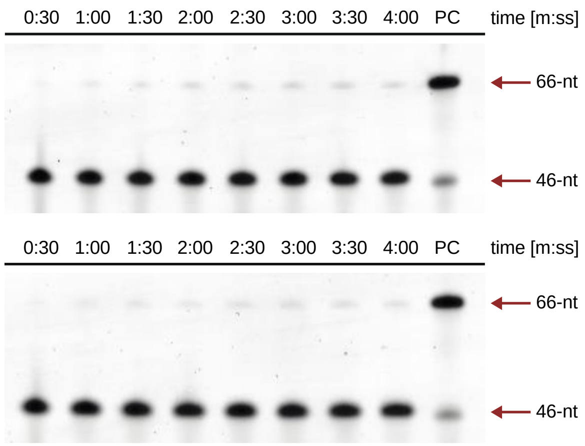

[CDP-Star] = 15.6 μM

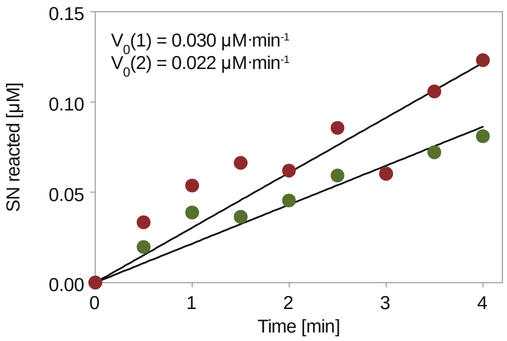

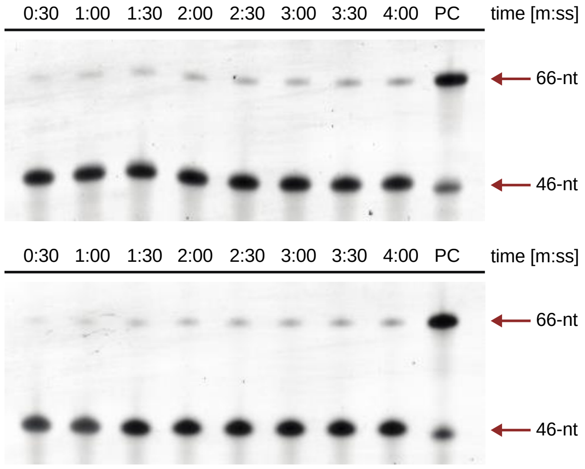

[CDP-Star] = 31.3 μM

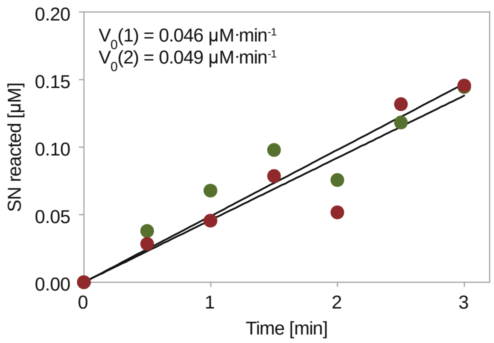

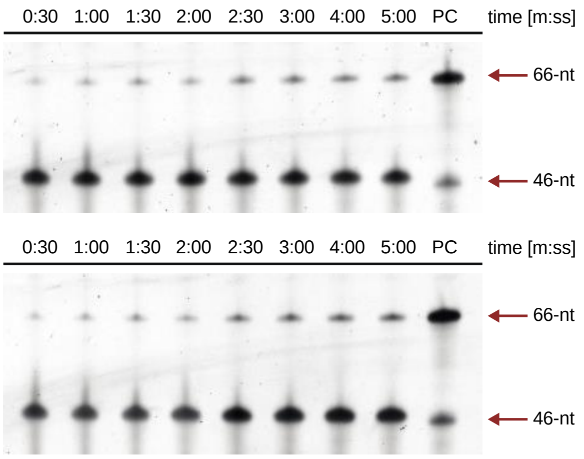

[CDP-Star] = 62.5 μM

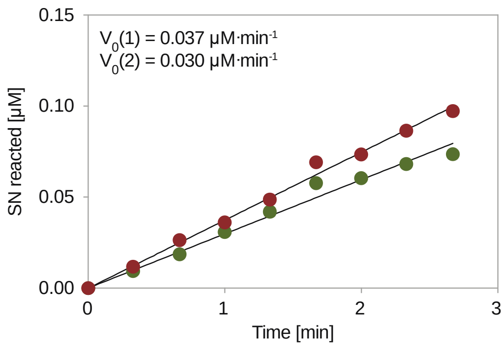

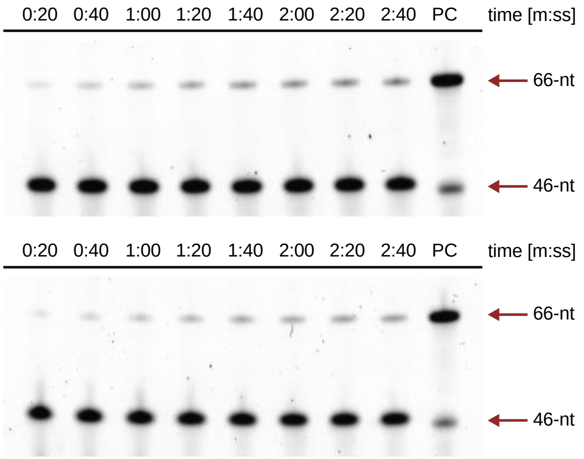

[CDP-Star] = 125 μM

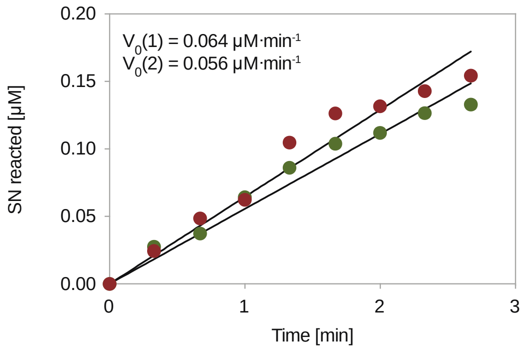

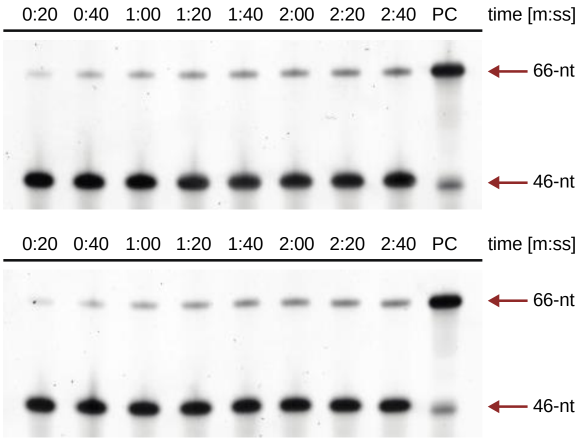

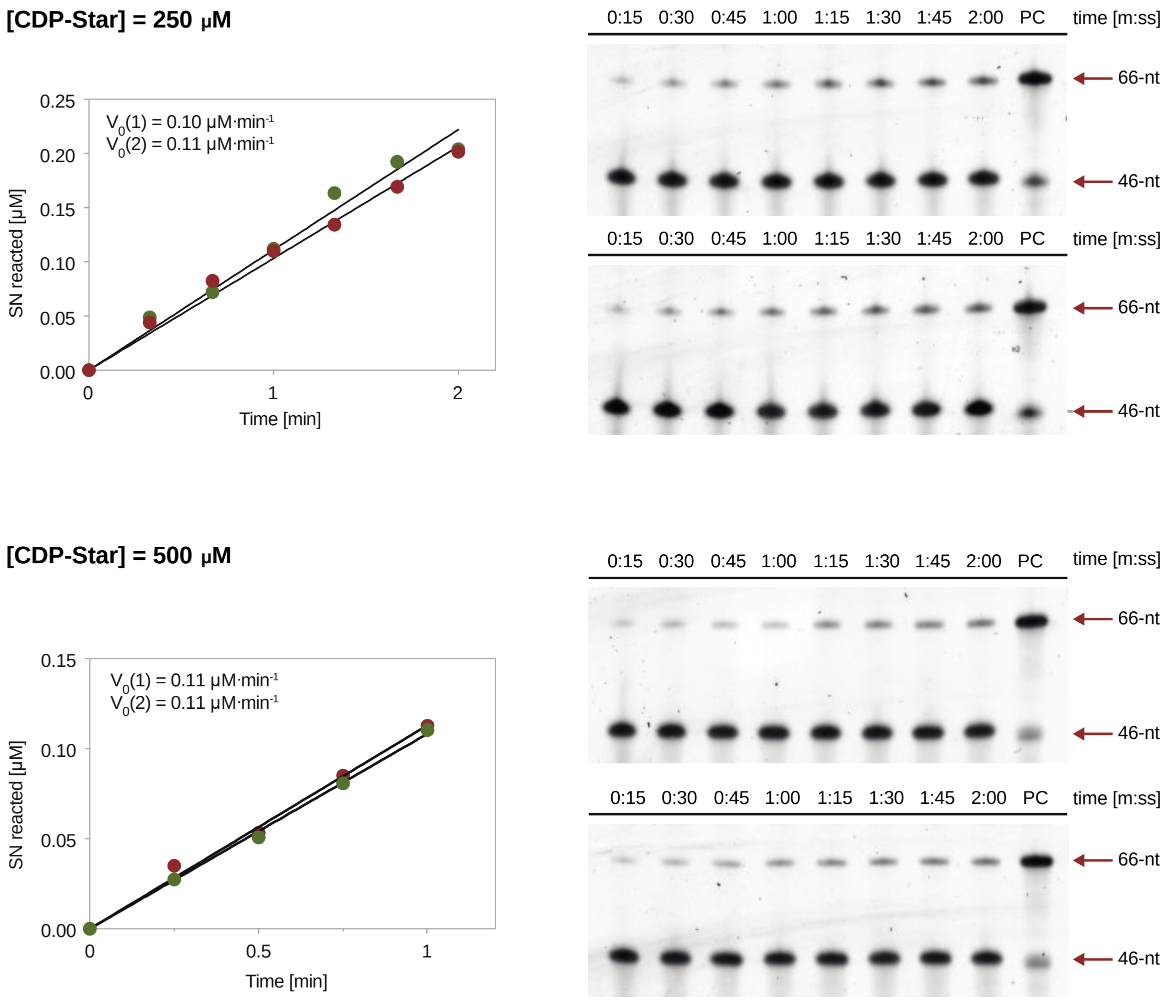

Figure S7. Determining the k_{cat} and K_m values of Supernova. Each panel shows the catalytic activity of Supernova at a different concentration of CDP-Star. Reactions contained 1 µM Supernova, and were performed in a buffer containing 50 mM HEPES pH 7.4, 20 mM KCl, and 1 mM $ZnCl_2$. Reactions were stopped at different times and, after tagging the reacted (5' phosphorylated) molecules by ligation, reacted and unreacted molecules were separated by PAGE. The positive control (PC) indicates a 5'-phosphorylated oligonucleotide that was used as both a control for the ligation reaction and a marker. Unreacted molecules are 46 nucleotides long whereas ligated ones have the size of 66 nucleotides (red arrows). Initial velocities were determined as described in the methods, and plotted as a function of CDP-Star concentration to determine k_{cat} and K_m values.

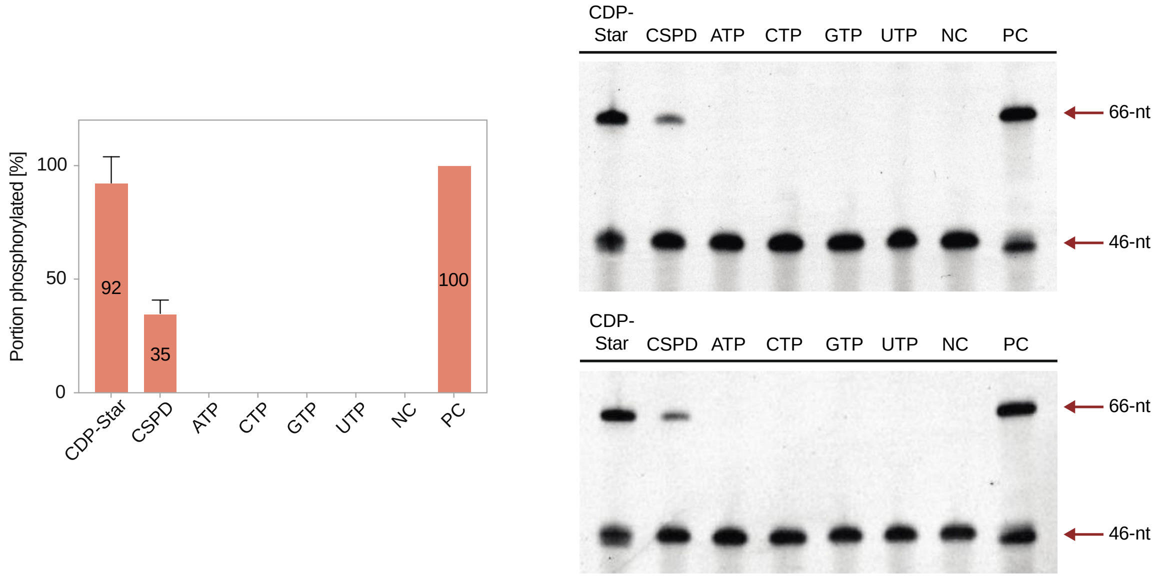

Figure S8. Substrate specificity of Supernova. Self-phosphorylation of Supernova in the presence of CDP-Star, CSPD (a substrate that differs from CDP-Star by the deletion of a single chlorine), ATP, CTP, GTP, and UTP. Reactions contained 1 µM Supernova and 100 µM substrate, and were incubated for 1 hour in a buffer containing 50 mM HEPES pH 7.4, 20 mM KCl, and 1 mM $ZnCl_2$. They were then analyzed using the ligation assay as described in Materials and Methods. NC indicates a reaction containing Supernova with no substrate while PC indicates a 5'-phosphorylated oligonucleotide that was used as both a control for the ligation reaction and a marker. Unreacted molecules are 46 nucleotides long while ligated ones have the size of 66 nucleotides (red arrows).

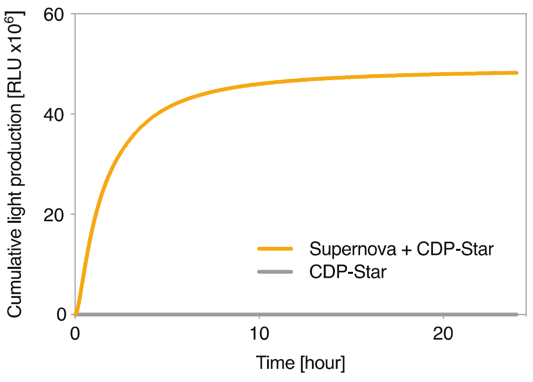

Figure S9. Light production of Supernova over 24 hours. Reactions were performed as in Figure 3b.

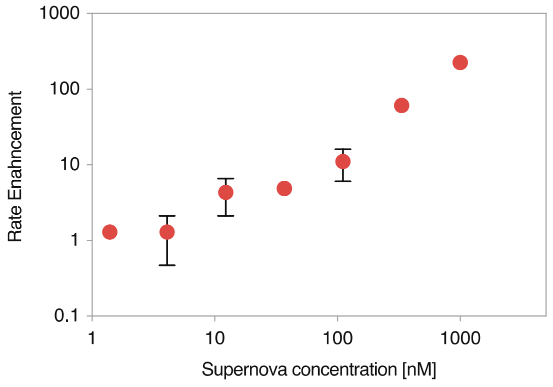

Figure S10. Limit of detection of Supernova. Light production at different concentrations of Supernova. Reactions contained 62.5 µM CDP-Star, and were performed in a buffer containing 50 mM HEPES pH 7.4, 20 mM KCl, and 1 mM $ZnCl_2$. Rate enhancements indicate the amount of light produced in the presence of deoxyribozyme divided by the amount of light produced in the absence of deoxyribozyme. Each point is the average of three measurements with error bars indicating the standard deviation.

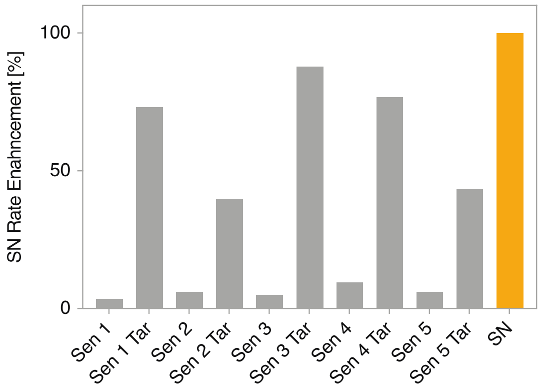

Figure S11. Catalytic activity of a light-producing oligonucleotide sensor. Light production of unmodified Supernova (yellow bar), and five Supernova sensors (grey bars; see also Figure 4c of the main text). Reactions contained 1 µM Supernova or Supernova sensor, 10 µM target oligonucleotide (or 0 µM for unmodified Supernova), and 62.5 µM CDP-Star, and were performed in a buffer containing 50 mM HEPES pH 7.4, 20 mM KCl, and 1 mM $ZnCl_2$. See Table S1 for more information about these sequences.

Supplementary Tables

Table S1. List of oligonucleotide sequences.

**Name**	**5' → 3' nucleotide sequence**
Supernova	identical to H2 core
Pool1	GGAAGAGATGGCGACN$_{70}$AGCTGATCCTGATGG
REV1	CCATCAGGATCAGCT
FWD1	GAATTCTAATACGACTCACTATA
FWD1r	GAATTCTAATACGACTCACTATrA
Splint1	GTCGCCATCTCTTCCTATAGTGAGTCGTATTAG
Pool2	GGAAGAGATGGCGACGACACAGGGACGATGCCGAATATCCTCAGTGCGCAGGGCCGCAGGGGGGAGTGACTTGGGATGGGGGG<br>TCCACTAATGATCTGCCCGATG<br>(underlined NTs were mutagenized at the rate of 21%)
REV2	CATCGGGCAGATCATTAGTG
FWD2	ACCGCTCAGGTGTAGTATCA
FWD2r	ACCGCTCAGGTGTAGTATCrA
REV2p	pCATCGGGCAGATCATTAGTG
Splint2	GTCGCCATCTCTTCCTGATACTACACCTGAGCGGT
H1	GGAAGAGATGGCGACGACACAGGGACGATGCCGAATATCCTCAGTGCGCAGGGCCGCAGGGGGGAGTGACTTGGGATGGGGGG<br>TCAGCTGATCCTGATGG
H1 core	GGAAGAAAAAGAATATCCTCAAAAGGGGAGTGACTTGGGATGGGGG
H2	GGAAGATATGGCGCGAACAAATGGACAATGCCGAATATCCCCCGCACGCAGGGCAACAAGGGGGAGTGACTTGGGATGGGGGCT<br>GCACTAATGATCTGCCCGATG
H2 core	GGAAGAAAAAGAATATCCCCAAAAGGGGAGTGACTTGGGATGGGGG
H3	GGAAGAAATGTAGAGGAAACAGTGACTCTGCAGAATATCCTCACTGCGTAGTGGGGCAGGGGGGAGTGACTTGGGATGGGGGCT<br>ACACTAATGATCTGCCCGATG
H3 core	identical to H1 core
Sensor polyA	GGAAGAAAAAGAATATCCCCAAAAAAAAAAGGGGAGTGACTTGGGATGGGGGAATTTTTTTTTTTTTTT
Target polyA	AAAAAAAAAAAAAAA
Sensor 1	GGAAGAAAAAGAATATCCCCCAATCGTGCGGGGAGTGACTTGGGATGGGGGAAGCACGATTGACCTTC
Sensor 2	GGAAGAAAAAGAATATCCCCCTCTTAAGAGGGGAGTGACTTGGGATGGGGGAATCTTAAGAGAAGGGA
Sensor 3	GGAAGAAAAAGAATATCCCCGGCACTGATGGGGAGTGACTTGGGATGGGGGAAATCAGTGCCCAGTGG
Sensor 4	GGAAGAAAAAGAATATCCCCATGATCGGAGGGGAGTGACTTGGGATGGGGGAATCCGATCATCCGAAG
Sensor 5	GGAAGAAAAAGAATATCCCCAGTAATAGCGGGGAGTGACTTGGGATGGGGGAAGCTATTACTTATCTT
Target Oligo 1	GAAGGTCAATCGTGC
Target Oligo 2	TCCCTTCTCTTAAGA
Target Oligo 3	CCACTGGGCACTGAT
Target Oligo 4	CTTCGGATGATCGGA
Target Oligo 5	AAGATAAGTAATAGC

Table S2. Bioinformatic tools used for data analysis.

Tool	Version	Source
cutadapt	1.15	https://cutadapt.readthedocs.io/en/stable/
fastq-join	1.3.1	https://github.com/brwnj/fastq-join
Clustal Omega	1.2.4	https://www.ebi.ac.uk/Tools/msa/clustalo/
FastQC	0.11.5	https://www.bioinformatics.babraham.ac.uk/projects/fastqc/
DiffLogo	2.8.0	https://www.bioconductor.org/packages/release/bioc/html/DiffLogo.html
ViennaRNA	2.4.12	https://www.tbi.univie.ac.at/RNA/

Table S3. Rate enhancements of light production of selected sequences.

Sequence name	CDP-Star concentration [μM]		
	**1000**	**250**	**62.5**
Starting Pool	1.1	1.7	1.1
H1	6.3	33.1	68.2
H1 core	2.3	9.8	24.1
H2	35.3	172.4	468.4
H2 core	6.7	38.9	106.4
H3	31.5	170.8	319.8
H3 core	2.3	9.8	24.1

Table S4. Analysis of clones after the initial selection. Rate enhancement of light production and the extent of deoxyribozyme phosphorylation were determined after incubating for one hour. Reactions contained 1 μM deoxyribozyme and 1 mM CDP-Star and were performed in selection buffer containing 50 mM HEPES pH 7.4, 200 mM KCl, 1 mM $ZnCl_2$, 1 μM $Ce(SO_4)_2$, 0.1 μM $PbCl_2$. Note that the deoxyribozymes which produce light the most efficiently under these conditions do not always phosphorylate themselves the most efficiently. This could reflect differences in the rates at which these deoxyribozymes release dephosphorylated CDP-Star after the self-phosphorylation reaction.

	**Rate enhancement of light production**	**Portion phosphorylated [%]**
H1	6.4	17.3
Clone 2	2.7	36.8
Clone 3	1.1	70.4
Clone 4	1.0	3.7
Clone 5	1.0	57.9
Clone 6	1.0	70.1
Clone 7	1.0	7.8
Clone 8	1.0	1.8
Clone 9	0.9	54.7
Clone 10	0.9	51.2
Starting pool	1.0	0
